# Amylene

**Published:** 1857-08

**Authors:** John G. Orton

**Affiliations:** Binghampton, Broome County, N. Y.


### ART. VI.— *Amylene.* By John G. Orton, M. D., Binghampton, Broome County, N. Y.

Prof. S. B. Hunt, M. D.:

Dear Sir,— As every recorded trial, I believe, which has been made in this country, with this new anæsthetic agent has resulted in an entire failure, it affords me much pleasure to report to you the following *successful* case of its administration:

On the 26th of June last, with the aid of the improved French inhaler, I administered about ℥iii of amylene to a strong robust patient, and effected complete insensibility in two minutes. The operation to be performed was one likely to give the anæsthetic a fair trial, namely, the removal of the large nail from an extensively diseased and highly sensitive toe. The patient manifested not the slightest uneasiness during the operation, but amused herself in an examination of the inhaling apparatus.

The pulse was but slightly accelerated, and no coughing nor vomiting ensued; in short, not a single unpleasant symptom manifested itself during or succeeding the administration.

I am strongly inclined to believe that the so-called samples of amylene which have been heretofore used by experimenters in this country, were not chemically the same as that used in Europe, and that they possess no anæsthetic properties whatever. I perceive that the surgeons of New York city have given it, as they say, "a fair trial," but without success.

The specimen of amylene which I used in the case here reported, was sent me from London, by a friend, Dr. Snow, of King's College Hospital, where, he informs me, he has used it successfully in 181 cases. I have been constantly in the habit of using the various agents commonly used for the production of anæsthesia, and I must confess that I was exceedingly pleased with the use of amylene in the instance recorded. One peculiarity was noticed, which is worthy of remark, namely, that almost immediately on the removal of the inhaling instrument from the patient sensibility returned, and the patient was able to rise from bed with scarcely any feeling of dizziness or unpleasant sensations of any sort whatever.

This case, as it is the *first successful* one which has been reported in this country, is of interest to the profession, as it reëstablishes the practicability of its administration, and the importance of obtaining a pure article.

I shall continue my experiments with amylene as occasion affords, and report to the profession accordingly.

Binghampton, July 6, 1857.

